# An Auto-Focusing Method in a Microscopic Testbed for Optical Discs

**DOI:** 10.6028/jres.105.046

**Published:** 2000-08-01

**Authors:** Xiao Tang, Pierre L’Hostis, Yu Xiao

**Affiliations:** National Institute of Standards and Technology, Gaithersburg, MD 20899-0001

**Keywords:** auto-focus, disc testbed, optical discs, standard deviation

## Abstract

An auto-focusing method in a digital image system is demonstrated that uses a standard deviation of pixel gray levels as a feedback signal. In this system, an optical microscope and a charge coupled device (CCD) camera are used to create clear pit images of optical discs. A dynamic focusing scheme is designed in the system-control software, which is able to eliminate environmental disturbances and other noises so that a fast and stable focus can be achieved. The method shows an excellent focusing accuracy. The performance and possible applications of this method are discussed. The test results for optical discs are given in this paper.

## 1. Introduction

In an optical vision system, an autofocusing element plays an important role. An objective lens collects light from the object and makes a clear image at the focal plane if the distance between the object and the objective lens is perfectly controlled to meet the focusing condition. A feedback signal is always needed for the controlling system. There are many kinds of feedback signal that can be used for this purpose in different systems. For example, an astigmatic field lens and a quad-detector (hardware) are used to create a focusing feedback signal in Compact Disc (CD) drives [1]. For digital image signals grabbed via a CCD camera, the feedback signal for autofocusing can be extracted from the image itself by software using Fourier transforms [2]. Actually, details and sharp edges in an image are associated with high spatial frequencies because they introduce significant gray-level variations over a short distance. After Fourier transformation, more high frequency components will appear in a well-focused image. These high frequency components can be used as a feedback signal. However there are some limitations in the Fourier transform method. For instance, two dimensional (2D) Fourier transform takes a matrix of (2*^n^* × 2*^m^*) pixels into account, where *n* and *m* are integers, but the image of interest usually does not exactly fit this requirement, so that we can not use information from all pixels in the image.

In this paper, a standard deviation (*SD*) value of gray levels from all pixels in an image is used as the feedback signal for autofocusing. In this method, the calculation of *SD* is easy and fast. Also we developed a dynamic focusing scheme in which all instabilities, such as disturbances from the environment and noise from the light source, can be canceled. Therefore, the focusing accuracy achieved can be as good as a few nanometers. The principles and the experimental set up will be described. As an example, an application of this method to an optical disc testbed and the results obtained will be discussed.

## 2. The Experimental Setup

**Fig 1:**
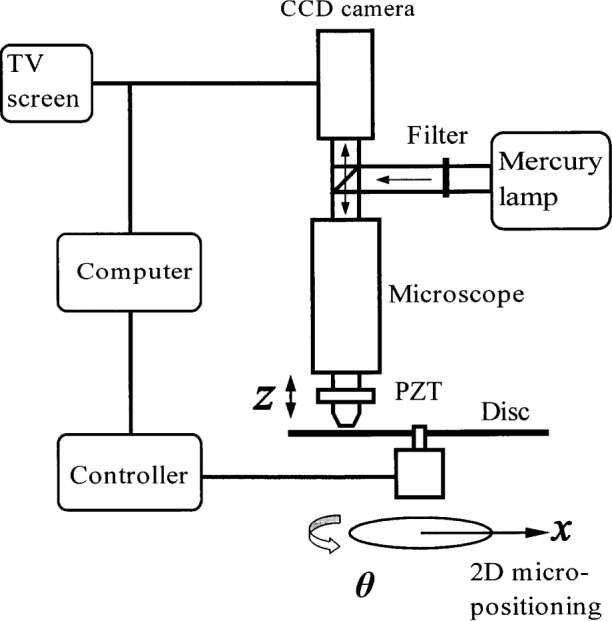
Schematic diagram of the experimental setup of the microscope testbed for the optical disc.

The auto-focusing system used in this experiment shown in Fig. 1 consists of an optical microscope, a CCD camera, a piezoelectric translator (PZT) actuator and a micro-stage for 2D (*x*, *θ*) movement. A personal computer (PC) controls the PZT actuator for focusing, moving the stage to look at different portions of the object, receives a digital image signal from the CCD camera, and then shows the image on its screen. The microscope uses a mercury lamp as the light source with an interference filter at 546 nm. The objective lens (× 100, numeric aperture (*NA*) = 0.8) can be adjusted manually as well as driven accurately by the PZT actuator. An adjustable collar cap is used on the objective lens for correcting the thickness of an optical disc under observation.

A CCD camera is used to acquire an image from a selected media layer where the information is recorded. The subsystem includes the image acquisition hardware as well as the software, which is developed in the Lab-VIEW^1^ environment, a graphical programming development tool. The LabVIEW software includes the features required for this application, such as libraries of functions for data acquisition and general purpose interface bus (GPIB).

Suppose the light source used in this experiment is not coherent but uniform enough within the numerical angle. Let us assume a very simple model for geometric analysis of our system. An image of a blank compact disc write once read many (CD-R) or compact disc rewrittable (CD-RW) disc consists of groove (dark) and land (bright) areas. Ideally, they should be in a shape with absolute sharp edges. In the real case, because of the diffraction limit, the image at the focal plane will have blurring, which means that there is a transition area Δ*X*_d_ between a dark area and an adjacent bright area in the image. From diffraction theory we estimate

$$\Delta X_{d}\approx \lambda /2(NA).$$

Here *λ* is the wavelength of the light source and *NA* is the numerical aperture of the objective lens. In the case of being off-focus, the objective lens has been shifted a distance Δ*Z* along the *Z* direction from the position where the system is exactly focused. This adds another blurring transition area Δ*X_z_*. We estimate this part as the following

$$\Delta X_{z}\approx 2(NA)\Delta Z.$$

To estimate the smallest focusing uncertainty Δ*Z*_acc_ that the system can reach, it is required that $\Delta X_{z}≦\Delta X_{d},$ therefore,

$$\Delta Z_{\text{acc}}≦\lambda /4{\left(NA\right)}^{2}.$$

We obtain the result that Δ*Z*_acc_ is equal to or less than 0.2 μm. In our experiment, a vertical stage driven by a stepping motor was used at the beginning. However, the controlling accuracy was not good enough to get repeatable results. A PZT actuator (nominal resolution 1 nm) is chosen to drive the objective lens. For disc movement in the *x* and *θ* directions, a translation stage and a rotational stage worked adequately.

A CCD camera is attached to the microscope to generate images. The image pickup area is 6.54 mm (horizontal) × 4.89 mm (vertical) with effective picture elements of 768 × 494 (horizontal × vertical) and 8 bit grayscale (black and white). The scanning frequencies are 15.734 kHz for the horizontal and 59.94 Hz for the vertical directions, respectively.

## 3. Software Development in Labview for System Control

LabVIEW runs on a PC. It controls the PZT as well as *x* and *θ* stages via a GPIB interface. It controls the *x* and *θ* stages to move the disc under the microscope. While the disc is moving, the CCD takes images of the pits in the data layer in the disc. In our case, each image consists of *n* = 640 × 480 = 307 200 pixels. Assuming that the *i*th pixel is at a gray level of *P_i_*, the mean gray level will be

$$\mu =\sum_{i=1}^{n}P_{i}/n.$$ (1)

The standard deviation *SD* is then simply defined as

$$SD={\left[\sum_{i=1}^{n}{\left(P_{i}-\mu \right)}^{2}/n\right]}^{1/2}.$$ (2)

**Fig. 2:**
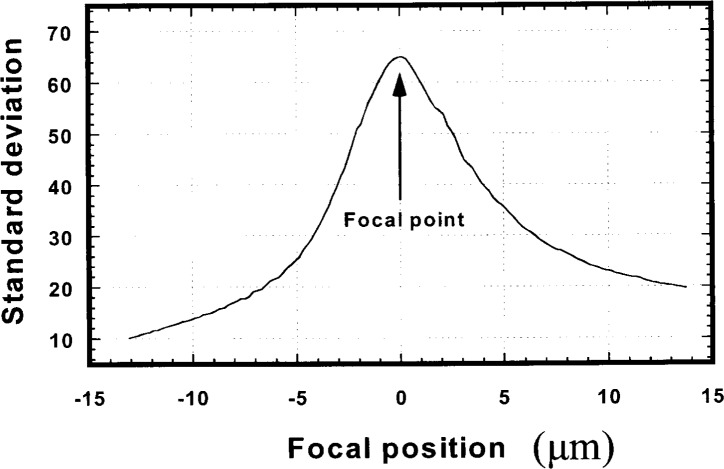
The curve of standard deviation for the disc image vs focusing position.

When the objective lens moves one step to a new position, the CCD camera grabs the image and sends the image signal to the computer. The computer then calculates *SD* for the image. Figure 2 shows a curve of *SD* vs focusing distance. One can see that *SD* is a maximum at the best focus position in the curve. To get the microscope focused, the algorithm should always try to find a larger *SD* until it reaches the maximum, then the microscope should lock on to it. However, if the system is out of focus at the beginning, the computer needs to know which direction, up or down, the PZT should move to approach the best focus position. We found that the mean gray level *μ* changes monotonically around the focal distance. This mean value was used to determine the direction of motion of the PZT. However, it was not stable because it changed when the intensity of the light source changed. We then turned to a dynamic focusing scheme.

In this scheme, our program lets the objective lens move just one small step (0.2 μm), up or down (randomly), and then compares the calculated *SD*_1_ (before moving) with *SD*_2_ (after moving). If *SD*_2_−*SD*_1_ > 0 (status a), then the objective lens moves in the same direction for another step. If *SD*_2_−*SD*_1_ < 0 (status b), then the objective lens moves in the opposite direction for another step.

At the beginning we manually adjust the objective lens to be near focus. The system will then automatically find a proper direction to move and keep moving (in status a). Finally, the system will reach the focus point and stay in status b. The algorithm will ensure that the objective lens moves around the focusing position, just one step up and another step down alternately. When the optical disc moves in the *x* direction or rotates, an off-focus situation usually arises, and the system is turned to status a until the best focus is again found.

## 4. Results and Discussion

**Fig. 3:**
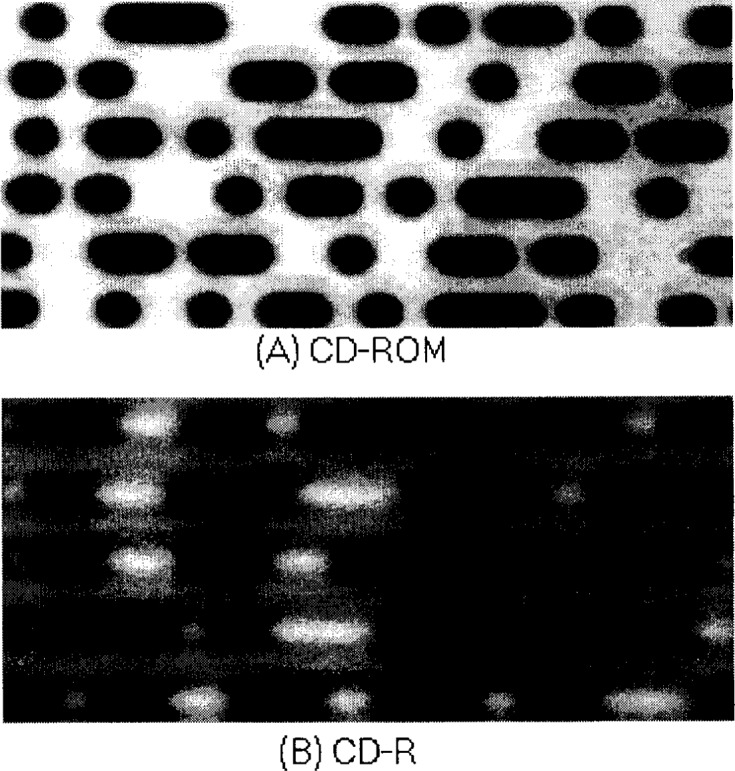
Sharp disc images taken at the focus position using the autofocusing system.

This method shows an excellent focusing accuracy. According to its specification, the resolution of the PZT is in the nm range. The focusing accuracy in this method is only one step of the PZT movement, which could be set as small as 0.01 μm. However, any step should not be less than Δ*Z*_acc_ as we discussed above. On the other hand, a larger step is more practical due to a faster focusing speed. A compromise value of 0.2 μm for one step is used in our experiment. With the testbed, sharp images of CD-ROM and CD-R discs can be automatically displayed on the screen (Figs. 3a and 3b).

For such a microscope system with high magnification, operational stability is a big challenge, especially when working in open air and with a relatively long operating time (a few hours, for example). Though a vibration-isolated optical table is used in our experiment, an on-focus status could be destroyed easily by other disturbances such as those due to an unstable light source or airflow from an air circulation duct. Our dynamic focusing scheme eliminates all these disturbances. In fact, the algorithm is designed such that the objective lens moves step by step without stopping. The focusing condition is checked by the system before each step is made. Then the focus condition is corrected immediately after each step. Thus this method is reliable and stable.

**Fig. 4:**
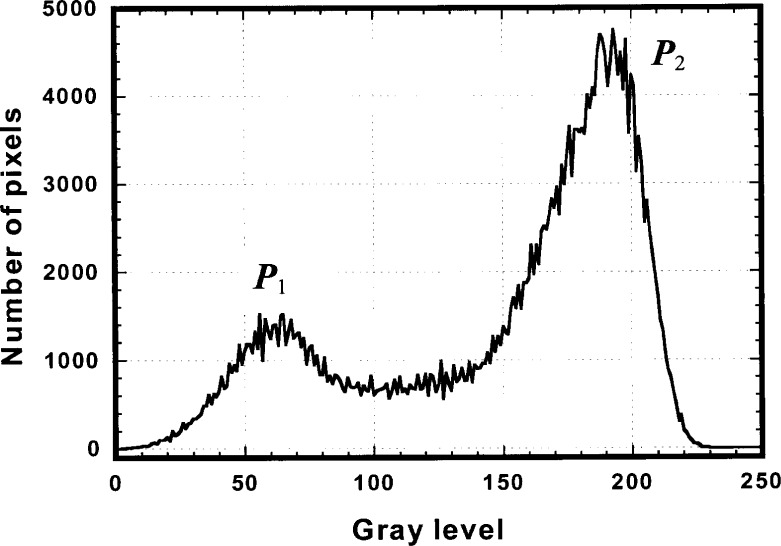
A typical histogram for a digital image of a CD-ROM disc recording layer, where *P*_1_ presents the dark where the information pits reside and *P*_2_ presents a bright area, which is the land area.

Figure 4 shows a histogram of the captured image from a CD-ROM disc. One can see there are two peaks, *P*_1_ and *P*_2_. *P*_1_ presents the dark area where the information pits reside and *P*_2_ presents a bright area, which is the land area. To evaluate the performance of the system, we simplify the model. Let us assume that there are *n*_1_ pixels presenting all pits and they are very dark. Their gray level *P*_1_ is 0 (the lowest). There are *n*_2_ pixels presenting the land area and they are very bright. Their gray level *P*_2_ is 255 (the highest), but we normalize it to 1. The total number of pixels in the image is *n* = *n*_1_ + *n*_2_. The average gray level is *μ* = (*n*_1_*P*_1_ + *n*_2_*P*_2_)/(*n*_1_ + *n*_2_). The standard deviation *SD* for this model image is then based on its definition.

$$SD={\left[\sum_{i=1}^{n}(P_{i}-u{)}^{2}/n\right]}^{1/2}={\left[n_{1}{\left(0-\mu \right)}^{2}+n_{2}{\left(1-\mu \right)}^{2}\right]}^{1/2}/n^{1/2}.$$ (3)

The larger the standard deviation, the larger the feedback control signal the system receives, so the better the control of the performance of the system. Considering that the definition of *μ* in Eq. (1) becomes

$$\mu =(n_{1}P_{1}+n_{2}P_{2})/n=n_{2}/n,$$

we take the derivative of *SD* with respect to *n*_1_ and then set it to zero to find the maximum value of the standard deviation. From this condition, we obtain

$$n_{1}=n_{2}.$$

That means for an image, if the dark and bright areas are almost equal, the system will have the best performance.

This method can be used on any digital image system without an additional hardware demand for generating a feedback signal. For example, it can be used in optical systems such as microscopes, telescopes, or digital cameras. It also can be used with other non optical systems such as electron microscopes if a digital image is the final output. This method may be suitable for on-site inspection of patterned product surfaces while the products are scanned under the camera.

## 5. Conclusion

We have demonstrated a new auto focusing method in a digital image system that uses the standard deviation of pixel gray levels as a feedback signal. A dynamic focusing scheme is designed in the system control software, which is able to eliminate environmental disturbances and other noises so that a fast and stable focus can be achieved. The focusing accuracy is demonstrated to be about 0.2 μm, although the potential uncertainty is as low as 10 nm. If the dark and bright areas are almost equal, the system will have the best performance. This method can be used in any digital image system without additional hardware demands for generating a feedback signal.

## Footnotes

1 Certain commercial equipment, instruments, or materials are identified in this paper to foster understanding. Such identification does not imply recommendation or endorsement by the National Institute of Standards and Technology, nor does it imply that the materials or equipment identified are necessarily the best available for the purpose.
